# Gifts of Time: Disrupting Dominant Temporalities in the Dementia Unit

**DOI:** 10.1002/hast.5000

**Published:** 2025-09-18

**Authors:** Hailee Yoshizaki‐Gibbons

**Keywords:** dementia, care, temporality, feminist disability studies, interdependence, clinical ethics

## Abstract

Within the dominant U.S. cultural imagination, dementia care is often constructed as a significant physical, mental, emotional, and financial burden, with a huge cost to individuals, families, and society. This cultural anxiety positions dementia as a signifier of dependence, helplessness, frailty, and loss. However, feminist disability studies offers a way to approach dementia and care as relational, collective, and political. Drawing on nine months of ethnographic research in the dementia unit of a nursing home, I illustrate how dementia care is a site of collectivity, resistance, and activism in a context of exploitation, control, and oppression. I uncover how institutionalized old women with dementia and immigrant and nonimmigrant women of color care workers navigate strict institutional routines, pressures of time management, and tightly controlled, predetermined care tasks and how they withstand these forces by making time for and giving time to one another, continuously (re)building relationships and investing in collective care that emphasizes interdependence.

Care—in the sense of concern and responsibility for meeting people's physical, emotional, and social needs and ensuring their health, well‐being, and safety—is an integral part of everyday life. Yet how people understand care can be complex, subjective, shifting, and contradictory. Care may represent a deeply close, intimate relationship, a strategy to survive and thrive, or a site of radical change, but it is also a highly regulated commodity and source of violence situated in interlocking systems of exploitation and oppression. As critical disability studies scholar Akemi Nishida explains, “Care is used to enforce top‐down dominant power as much as care is exercised at the grassroots level to enable resistance against such dominant power and enact transformative power for a more just world and way of living.”^1^

For people with dementia, care may be a particularly convoluted experience. While it may be meaningful, positive, and even empowering, it may also be fraught with concerns about encumbering loved ones, draining systems of resources, and being institutionalized in a nursing home or similar long‐term care facility. Within the dominant U.S. cultural imagination, dementia represents increasing dependence, helplessness, and frailty. The individual and cultural anxiety around the presumed loss of self that accompanies memory loss leads to isolation, erasure, and dehumanization for people with dementia. Accordingly, dementia care is often constructed and experienced as a significant physical, mental, emotional, and financial burden, with a huge cost to individuals, families, and society.

An added complexity to dementia care is the increasing commodification of care. Historically, older adults with dementia have been situated as “recipients” of care, with family, friends, and care workers serving as the “givers” of care.^2^ However, people with dementia are now increasingly described as “consumers” of care, with the care workers serving as the “providers.” One explanation for the turn to “consumer” is that disability and dementia rights movements have used the term to indicate that the person receiving services has autonomy and control over their care.^3^ However, the nursing‐home industrial complex, or the network of nursing homes; medical and pharmaceutical companies; device, linen, and food suppliers; and other entities that benefit economically from the segregation of old and disabled people in care facilities,^4^ also uses the term “consumer,” signifying that nursing‐home residents are sources of revenue.^5^ As the vast majority of nursing homes in the United States are for‐profit entities, this positioning reflects a neoliberal, capitalist approach to care, in which care is a product or good to be sold, purchased, and consumed. Whereas the recipient‐giver division of care suggests that the care worker is ranked above the person with dementia, the consumer‐provider ideology of care suggests that the person with dementia is ranked above the care worker. While distinct, both views of care are hierarchical and position the person with dementia and the care worker as opponents in a power struggle, obscuring the reality that both parties are subject to the same apparatuses of power: the institution and state.

In my research examining the care relationships between old women with dementia and the immigrant and nonimmigrant women of color employed to care for them, I have observed myriad ways in which institutional and state policies and practices were central to establishing care as a source of oversight, bodily management, and profit embedded in multiple systems of domination.^6^ The state and broader nursing‐home industry have tremendous power over both the old women with dementia and the care workers, and they have exerted that power to create the conditions of care that support the survival of the old women with dementia and care workers while also isolating, marginalizing, exploiting, and dehumanizing them.

However, drawing on feminist disability studies’ theories of interdependence and temporality, I also found that the two parties in the care dyad engaged with one another in ways that disrupted these power dynamics. Feminist disability studies integrates critical disability theory with feminist theory to understand how disability and the bodymind are situated in intersecting systems of power while concurrently serving as sites of solidarity and coalition.^7^ This approach examines the collective and political dimensions of care through concepts like interdependence, which concerns the ways that people rely on each other to survive and, ideally, thrive. A central aspect of interdependence is giving time to one another or making time for each other in ways that support each person's needs. In the dementia unit, I regularly witnessed this practice—which I term “gifts of time”—as an investment in care and connection in a temporal context defined by control, surveillance, and confinement.

Furthermore, gifts of time counter two key dominant cultural narratives about dementia care in nursing homes. The first cultural narrative that is challenged is that care in such contexts is one‐sided and hierarchal, with care workers positioned as providers of care and old people with dementia as receivers or consumers of care. The second contested cultural narrative is that nursing homes are exclusively places of decline and death, in which inhuman care is provided solely to keep residents alive and lacks affection or compassion. Such narratives lead to the assumption that old people with dementia and care workers are merely victims of their circumstances, subject to the total control of the institution and lacking true connection. Gifts of time reveal that old people with dementia and care workers create their own forms of meaning and kinship, subverting the institution and state. The resistance exerted by these individuals leads us toward imagining and creating better futures for dementia care.

## Care in the Dementia Unit

In the dementia unit of Cedarwood Care Center,^8^ institutional and state regulation, surveillance, and control deeply shaped the care relationships between older adults with dementia and direct care workers. One of the core ways these methods of disciplinary power were implemented was through time. I observed two common forms of dominant temporalities, or approaches to structuring time that are grounded in institutional and cultural power, that shape life and work in the dementia unit. These temporalities take the form of what I call “institutional time” and “bureaucratic time.”

At the core of long‐term residential care is the management of bodies via institutional time.^9^ This oversight is accomplished by implementing rigid routines, promoting strict time management, and executing predetermined care tasks that must occur at certain times or in a specific amount of time. For example, in the unit I observed, toileting was to occur approximately every two hours for each resident. Dressing was to occur in the morning, after each resident woke, and in the evening, before each resident went to bed. Feeding was to occur during each of the hour‐long mealtimes: breakfast (7:00 a.m. to 8:00 a.m.), lunch (11:30 a.m. to 12:30 p.m.), and dinner (4:30 p.m. to 5:30 p.m.). Transporting was to occur before and after each meal. Recording vital signs was to be done once every eight hours. Medications were to be distributed during mealtimes, prior to bed, or at both times, depending on the prescribed dosage (for example, every four or every eight hours). Though care workers are forced to follow institutional time and provide care around these temporal restrictions, old women with dementia are the primary subjects of institutional time, as their bodies are the ones being cared for and controlled by the nursing home.

Bureaucratic time is concerned with administering care to as many people as possible, operating the nursing home as a business, and ensuring compliance with governmental regulations. Bureaucratic time arranges staff shifts, measures the amount of time worked by care staff, establishes hourly or biweekly pay for care workers, monitors care staff's breaks, and determines whether specific outcomes required by the state were achieved (for instance, if residents were toileted at least once every two hours). Care workers are the primary subjects of bureaucratic time, as their labor is exploited to establish cost‐effective and profitable systems of care.

These dominant temporalities established the parameters for care in the dementia unit and were heavily influenced by the increasing economization of care, governmental regulations, the larger nursing‐home industry, and cultural beliefs about the value of old women with dementia and those who care for them. Old women with dementia and care workers have been valued primarily for their vulnerable positionality, which allows institutions to exploit them to earn profit. Beyond this, cultural ideologies render the care dyad invisible, marginalizing its members in ways that minimize or even erase their existence, their struggles, and their joy.^10^ At the same time, institutionalized older women with dementia and women of color care workers navigate and withstand these forces by making time for and giving time to one another, and through these gifts of time, they continuously (re)build relationships and invest in collective care that emphasizes interdependence.

## Gifts of Time: Giving and Making Time in the Dementia Unit

The more time I spent observing interactive moments between old women with dementia and care workers in the dementia unit of Cedarwood Care Center, the more I began to question the meaning and significance of “giving time” and “making time.” Despite knowing and using these idioms for years, I had never given them much thought. According to *Merriam‐Webster*, giving time means to “use one's time and effort to help others,” and making time refers to “[causing] an amount of one's time to be available to do something for or with someone.” Both idioms are based on the cultural assumption that time is valuable and hence it is meaningful and important to give or make time to help, do something with, or do something for another person. Furthermore, these idioms underscore that time is a central part of care. The physical, emotional, and social aspects of care that people provide to each other are temporally bound—they require time and frequently involve repetition, duration, synchronizing, and routine.

In the dementia unit of Cedarwood Care Center, institutional and bureaucratic time created a temporal economy in which time was a valuable and scarce resource. Neither the old women with dementia nor the care workers had much autonomy or choice regarding how they “spent” their time in this temporal economic context. But even as their time was tightly controlled, acts of giving or making time—gifts of time—unsettled the dominant temporalities in ways that allowed for shared moments of connection. I frame these moments as gifts because gifting, as a cultural practice, represents a commitment to creating and maintaining relationships, affirming others’ worth, acting in a spirit of generosity, and establishing a sense of belonging. For instance, Stella, a care worker, emphasized the importance of making time for and giving time to people with dementia when they were in emotional unrest or turmoil.

I asked her, “What do you do when you see that [one of the old women with dementia is in distress]? What do you try to do to assist them?”

She responded, “I spend time with them. I know I have thirteen other people, but in a case like that, I feel that's how I deal with my residents. To me, that's an emergency. So, I spend more time with them. I'll get them comfortable, you know, and I just spend more time with them.”

“So, you give them one‐on‐one attention?” I asked.

“Give them the one‐on‐one even though I don't have the time, but I make the time,” Stella explained. “Being a one‐to‐one person, if I see one of my residents [is] upset or something like that, I try to accommodate them—no matter how long it takes.”

Although care, particularly in institutions, is typically understood as one‐sided and hierarchal, the old women with dementia sought to make time for and give time to the care workers. While the residents did not experience the same pressures and were likely unaware of the regulations and restrictions defined by the state and the administration of Cedarwood Care Center, many of them were aware of how strained and pressed the care workers were while trying to complete their work. Given this, the old women with dementia made time to acknowledge how busy the care workers were and thank them, even for everyday tasks the care workers might be “expected” to do, as when Eleanor, a resident, responded to care from Serena.

Eleanor was really enjoying her juice at dinner and finished it quickly. Serena noticed and brought her more juice. “Oh!” Eleanor said, pleasantly surprised. “Oh, thank you, my dear!” Eleanor then turned to another old woman with dementia at her table, Marlene. “She did that for me!” Eleanor told her. “I appreciate that!”

Marlene shared in Eleanor's happiness over this simple gesture of kindness. “Yeah, she did! There you go, kiddo!” Serena smiled.

While these small gestures of gratitude may seem minor or even insignificant, they were important. The care workers were thankful for the residents’ expressions of appreciation, especially in a line of work where they often felt invisible and undervalued. Ciera, a care worker, talked about the importance of this appreciation, telling me, “Nobody appreciates you here [at Cedarwood Care Center]. But the residents.”

“How do the residents show appreciation?” I asked.

“Well, they always smiling!” Ciera answered. “Or some can verbally say thank you. Bernard says thank you all the time. Fauna will. Vladimir and his wife say thank you in Russian. Or you know, they will just touch your hand. Give you a hug. Give me a kiss, kiss my hand.”

I remarked that Ciera and I had talked about how “this is a stressful job; it's hard work.” I asked, “What makes your job feel ‘worth it’?”

“Providing care for the residents,” Ciera responded. “Making the residents feel good. I try to treat my residents like my family. Seriously. And I think just them, them appreciating it. Appreciating you.”

The old women with dementia would also give time to the care workers by trying to assist them with their jobs in other ways. I saw this, for example, with Bernice. At dinner, Bernice's assigned certified nursing assistant, Guadalupe, suddenly shrieked, “Bernice! Where is Bernice?” Bernice's empty wheelchair was at her table, but Bernice was nowhere in sight. “Oh, my God, where is she?” Guadalupe exclaimed.

Everyone began looking around. “There she is!” The private caregiver of one of the other residents pointed. Bernice was standing at one of the back tables, stacking dishes and cleaning the table. Guadalupe laughed a little, clearly relieved and a little touched by the gesture.

Another instance was one in which one resident stepped in by comforting another. Laurelle was often restless and would repeatedly try to stand and walk without support. However, Laurelle was less likely to do so when someone was with her and making her feel cared for. The care workers tried to do this when they could, but often they were helping others. Patricia, another resident, sat next to Laurelle and rubbed her back, calming her. Meera, a care worker, praised her: “Thank you, Patricia, thank you. You are so sweet; you help so much. Everybody.”

In these moments, the old women with dementia approached care as collective and communal, rather than as a hierarchal, transactional relationship in which they were the receivers or consumers of care and the care workers were the providers of care. By giving time to and making time for each other, the old women with dementia and the care workers became partners in care. Although they were not equal partners due to their differing social locations and distinct experiences of marginalization and exploitation,^11^ the care dyad challenged the dominant temporalities of the dementia unit through small gifts of time. Making time and giving time became experiences of resistance, survival, and even joy. Moreover, through giving and making time, old women with dementia and care workers made investments in their relationships—an aspect of institutional care that is uncompensated, overlooked, and undervalued.

The scarcity of time in the dementia unit constructed even small gifts of time, such as the following moments, as extraordinarily valuable:

During one gift of time, I was sitting in the common area of C‐wing with the residents, next to Laurelle on the love seat. The news was on, but no one was really watching. Many of the residents were asleep. Evie was watching the residents, and Karina, who was assigned to care for the residents who live in C‐wing, hustled about, toileting residents; changing any clothing soiled with food, urine, or excrement; and ensuring that the residents had fresh towels and linens in their rooms. Laurelle suddenly announced, “I am thirsty!”

Since Evie had to watch and could not leave the residents, I went to the kitchen and fetched Laurelle a glass of water. I brought it back, and Laurelle took a sip and said, “That's not good! I want something good to drink!” Evie offered Laurelle a milkshake from the daily snack tray, and Laurelle drank it but then asked for more. Evie told her each resident got only one; there were no more. “I want something good to drink!” Laurelle repeated.

Suddenly, Karina, Laurelle's certified nursing assistant, appeared. I noticed that Karina had gone to the kitchen and picked up Laurelle's personal cup that had a lid to prevent spills and a straw. “I have something good to drink!” she announced triumphantly, handing the cup to Laurelle. Laurelle took a sip, and her face lit up.

Karina smiled and winked at me, whispering that it was carbonated water.

“Where did you get this?” Laurelle asked, surprised. “From my cousin?”

“From your fridge! [Your son] brought it for you!”

“Ah, yes!” Laurelle replied gleefully, taking another long drink. Karina explained that Laurelle didn't care for tap water but loved carbonated water.

In another case, it was dinnertime. Sylvia, a resident, noticed Trishna hurrying by with a cartful of plates on her way to serve residents at one of the tables in the back of the dining room. “You are so pretty!” Sylvia called out.

Trishna served the residents and then quickly returned to Sylvia's table. “Thank you so much, Sylvia! You are so pretty too!” She stroked Sylvia's face lovingly.

“Oh, thank you!” Sylvia smiled. “You are welcome!” Trishna responded, taking Sylvia's hand in hers. Sylvia patted her hand.

A few minutes later, Ashanti, another certified nursing assistant, served Sylvia her food. “Thank you, sweetheart!” Sylvia said to her.

“Oh, Sylvia, I love you,” Ashanti responded. “Give me a hug!” Ashanti and Sylvia hugged, looked into each other's eyes, and smiled.

If a casual observer or infrequent visitor to the dementia unit had witnessed these interactions, they may have interpreted them in terms of Karina simply “being nice” or even “just doing her job” and Sylvia being “a sweet old lady.” However, after immersing myself in the dominant temporalities of the dementia unit, alongside the care workers and old women with dementia, I noticed the significance of these gifts of time. Understanding the economy of time at work in the dementia unit transformed kind yet seemingly mundane and minor gestures into gifts of time—meaningful moments of care and interdependence.

## Gifts of Time and Liberatory Futures

The pressures of institutional and bureaucratic time led to systems of care that did not encourage or outright prevented deep connection between the old women with dementia and care workers, and it is important to acknowledge that these care relationships were not perfect or ideal. As with all relationships, they were complicated and, at times, outright messy, such as when the care workers were unable to meet the needs of the old women with dementia, the old women with dementia treated the care workers like “the help,” or the old women with dementia were infantilized. These complexities were amplified by the institutional care context, which operated on the exploitation and marginalization of these two distinct yet interconnected groups of multiply marginalized women. Their interdependence was, in many ways, structural: the members of the care dyad needed each other to survive.

However, the old women with dementia and care workers were able to create and maintain care relationships that aligned with feminist disability studies politics of relationality, interdependence, and temporality by centering collectivity, shared moments of connection, and making time for and giving time to each other, even in a context of institutional and state power, exploitation, and violence.^12^ In doing so, the old women with dementia and care workers challenged dominant cultural narratives about dementia care, which construct it as hierarchal, one‐sided, and defined by decline, loss, and death. The care dyad is writing new stories of dementia care—defined by resistance, joy, and kinship. In this regard, gifts of time in the dementia unit are part of Alison Kafer's vision for “more accessible futures” and a future of dementia care that is grounded in justice and love.^13^

Furthermore, gifts of time may be understood as a liberatory approach. The economist Mike Konczal observed, “Time is the universal measure of freedom,” referring to how control over one's time is a form of emancipation in a capitalist society.^14^ While the gifts of time described above may be overlooked, devalued, and disregarded by the institution and state, this is precisely why they are so powerful. They allow the old women with dementia and care workers to engage in a collective reimagining of care outside the confines of the institution and the state. Taking these acts of care seriously as resistant temporalities moves us toward a future in which care is not a site of exploitation, violence, and confinement but is instead accessible, liberating, empowering, and central to radical political and social transformation.

## Acknowledgments

This research was funded by the Dean's Scholar Fellowship, Alice J. Dan Dissertation Award, and Charlotte A. Tate Award for Multidisciplinary Research at the University of Illinois at Chicago and the Philanthropic Educational Organization (P.E.O.) Scholar Award.
